# Smith-Magenis Syndrome—Clinical Review, Biological Background and Related Disorders

**DOI:** 10.3390/genes13020335

**Published:** 2022-02-11

**Authors:** Berardo Rinaldi, Roberta Villa, Alessandra Sironi, Livia Garavelli, Palma Finelli, Maria Francesca Bedeschi

**Affiliations:** 1 Clinical Genetics Unit, Fondazione IRCCS Ca’ Granda Ospedale Maggiore Policlinico, 20122 Milan, Italy; berardo.rinaldi@policlinico.mi.it (B.R.); roberta.villa@policlinico.mi.it (R.V.); 2 Experimental Research Laboratory of Medical Cytogenetics and Molecular Genetics, Istituto Auxologico Italiano, IRCCS, 20145 Milan, Italy; a.sironi@auxologico.it (A.S.); finelli@auxologico.it (P.F.); 3 Department of Medical Biotechnology and Translational Medicine, Università degli Studi di Milano, Segrate, 20090 Milan, Italy; 4 Clinical Genetics Unit, Azienda USL-IRCCS of Reggio Emilia, 42123 Reggio Emilia, Italy; livia.garavelli@ausl.re.it

**Keywords:** Smith-Magenis, SMS, *RAI1*, 17p11.2 deletion syndrome, sleep disorders

## Abstract

Smith-Magenis syndrome (SMS) is a complex genetic disorder characterized by distinctive physical features, developmental delay, cognitive impairment, and a typical behavioral phenotype. SMS is caused by interstitial 17p11.2 deletions (90%), encompassing multiple genes and including the retinoic acid-induced 1 gene (*RAI1*), or by pathogenic variants in *RAI1* itself (10%). *RAI1* is a dosage-sensitive gene expressed in many tissues and acting as transcriptional regulator. The majority of individuals exhibit a mild-to-moderate range of intellectual disability. The behavioral phenotype includes significant sleep disturbance, stereotypes, maladaptive and self-injurious behaviors. In this review, we summarize current clinical knowledge and therapeutic approaches. We further discuss the common biological background shared with other conditions commonly retained in differential diagnosis.

## 1. Introduction

Smith-Magenis syndrome (SMS; OMIM #182290) is a rare genetic disorder characterized by developmental delay (DD)/intellectual disability (ID), typical behavioral characteristics, distinct facial features evolving with age, and multiple congenital anomalies [1,2,3]. 

The first report of SMS was in 1982 by Ann CM Smith who described two individuals with an interstitial deletion of the p11 band on chromosome 17 [4]. Four years later, Ann Smith and Ellen Magenis expanded the previous work including nine unrelated patients and delineating the phenotypic spectrum of this condition [2], later named SMS. 

The syndrome is caused either by the recurrent 17p11.2 deletion or pathogenic variants in *RAI1* causing its haploinsufficiency [2,5]. The majority of subjects carry a 17p11.2 deletion whereas the remaining ones show a pathogenic variant in *RAI1* [6]. *RAI1* encodes a protein that acts as transcriptional regulator and is involved in neurodevelopment, behavioral function, and circadian rhythms, such as the sleep–wake cycle.

Almost all patients reported to date are sporadic, although rare familial clustering has been described as well. Since the identification of *RAI1* as the major gene of SMS [5], an increasing number of pathogenic variants have been detected in individuals presenting with the core phenotype of SMS and no 17p11.2 deletion.

The birth incidence is estimated to be between 1:15.000 [7] to 1:25.000 [3], with no predominance of either sex [8]. 

This review focuses on the current knowledge of the clinical features of SMS, with particular attention to behavioral problems and their treatment options, as well as the molecular aspects providing an overview of the spectrum of SMS-related disorders.

## 2. Clinical Features

### 2.1. General Appearance

**Figure 1:**
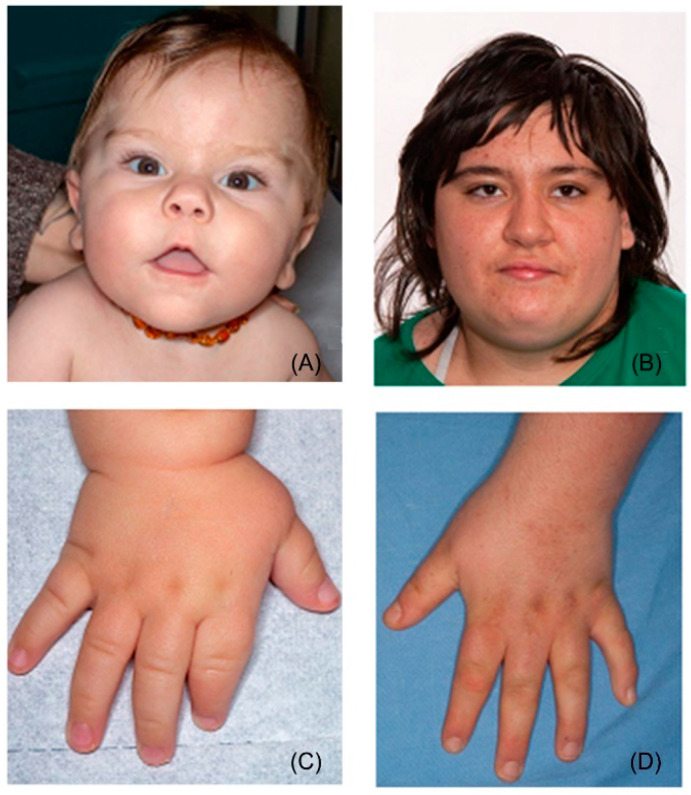
Main clinical anomalies in SMS: Classic facial phenotype in child (**A**) and in adolescence (**B**). Typical small hand with brachydactyly (**C**,**D**).

The facial appearance could be enough recurrent and specific to prompt a gestalt recognition (Figure 1A,B). Typical dysmorphisms include brachycephaly (HP:0000248), broad face (HP:0000283), frontal bossing (HP:0002007), synophrys (HP:0000664), upslanting palpebral fissures (HP:0000582), deep-set eyes (HP:0000490), midface retrusion (HP:0011800), depressed nasal bridge (HP:0005280), short and broad nose (HP:0003196, HP:0000445), low-set and/or abnormally shaped ears (HP:0000369, HP:0000377), everted and tented upper lip vermilion (HP:0010803, HP:0010804), prognathism (HP:0000303) [1,2,3,9]. Other signs reported less consistently are thick hair with sparse temporal scalp distribution, thick eyebrows, eyelash trichomegaly, telecanthus/hypertelorism or hypotelorism, epicanthus, strabismus, cleft lip and/or palate, downturned corners of mouth, micrognathia (during infancy) [1,2,8,9,10,11,12,13,14]. The facial traits become progressively more pronounced from childhood to adulthood, particularly due to the disproportion between the midface retrusion and the increasing width and protrusion of the mandible, emphasizing prognathism [1,10,15]. This transition illustrates also why facial dysmorphisms of SMS may recall Down syndrome in infancy (brachycephaly, broad face with midface retrusion, upslanting palpebral fissures, short nose) [1,15] and fragile X syndrome in adulthood (prognathism) [16]. Dental development is often affected, leading to malocclusion, taurodontism, and teeth agenesis, especially of the second lower premolars [17]. The voice may be peculiar and is usually described as hoarse, low-pitched, and raspy [2,3,18,19,20]. Hands and feet are broad, short, and associated with brachydactyly (Figure 1C,D). Clinodactyly of the fifth fingers, 2–3 toe syndactyly, single transverse palmar crease, and finger pads may also be present; polydactyly has been reported twice [2,21,22,23,24,25]. Common dermatological features comprise xerosis, folliculitis of the back, acral pachydermia, and plantar keratoderma [12]. Some authors have noticed that the pigmentation of hair and skin of SMS individuals is lighter than that of their relatives [12,15].

### 2.2. Growth

At birth, auxological parameters usually fall within the normal range [2,15,25]. The evolution of weight tends to be biphasic: in the first years of life, feeding difficulties may even cause failure to thrive whereas, from preadolescence onwards, overeating, low daily activity, and possible iatrogenic side effects often cause a significant ponderal gain [2,3,15,25,26]. From the age of 9 years, about half (47.8%) of individuals have a weight > 95th percentile [27]. Stature is usually below the normal range in toddlerhood and early childhood while it partially recovers with age [3,21]. Most of available data concerning height derive from series enriched in individuals in their middle/late childhood, so that the high prevalence of short stature (range 66% [28]–81% [3]) might not reflect the final height, which should place in the low percentiles of the normal range [29]. Moreover, a recent retrospective study found short stature to be less frequent than previously thought (25%) [25]. When addressing adults with SMS, short stature is reported to be present in 5–10% of them [15]. Moreover, both height and weight vary accordingly to the underlying genetic defects. In particular, short stature is more documented in patients carrying the 17p11.2 microdeletion than in those with *RAI1* pathogenic variants (81% vs. 10%) [8]. Short stature is not usually due to an impaired functioning of the growth hormone (GH) axis. The total secretion of GH has been proved to be normal, although the peak values were lower than those of controls [30]. On the other hand, a GH deficit was identified three times [25,31,32] and replacement therapy might have proved to be effective for the final stature only once [32], requiring further studies to elucidate its real benefit. Microcephaly is not common and varies from 16% [25] to 37.5% [33]. 

### 2.3. Multisystemic Manifestation

*Cardiovascular.* The frequency of congenital heart defects (CHDs) varies from 25% [34] to 45% [28]. The most frequent CHDs are atrial septal defects, ventricular septal defects, tetralogy of Fallot, valvular abnormalities, and total anomalous pulmonary venous connection [34]. Arrhythmias and conduction disorders are present in 12% of subjects and include right conduction delay and ventricular pre-excitation [9,34]. Importantly, echographic signs of dysfunction of both the right and the left ventricle have been detected in all the patients of a cohort of 24 SMS individuals, raising concerns about the possible occurrence of heart failure [34].

*Genitourinary* malformations are reported in 14% [8]–35% [9] of subjects. The following abnormalities have been pointed out [2,9,25,28,35,36,37,38,39]: unilateral renal agenesis, ectopic kidney, abnormally small or hypertrophic kidney(s), renal dysplasia, duplex collecting system, renal pelvic ectasia, ureteropelvic junction obstruction, ureterovesical junction ectasia or obstruction, vesicoureteral reflux. Male genitals may present cryptorchidism, testicular ectopia, shawl or underdeveloped scrotum, micropenis [2,25,33,40].

*Otolaryngologic* domain is consistently affected in SMS [9,10,28]. A specific hearing assessment performed on 97 individuals detected an overall presence of hearing loss in at least one ear in 78.9% of the cohort, the majority of them demonstrating a slight or mild impairment. Sensorineural hearing loss was the most represented, its occurrence increased in older subjects and longitudinal data revealed a progressive decline with age. On the contrary, the proportion of conductive hearing loss was higher in childhood, corresponding to a higher percentage of flat tympanograms, in turn presumably due to middle ear effusion [41]. Indeed, acute and chronic otitis media are common in children and often require ventilation tubes [10,29]. In addition to hearing loss, also hyperacusis is part of SMS and has been estimated to be as frequent as 73.5% by means of a questionnaire addressed to caregivers [41]. Velopharyngeal insufficiency (VPI) is recurrent and has been related either to the hypotonia of the orofacial district or to patent or submucous palate cleft [9,16,28]. VPI may lead to hypernasal speech, phonological errors, and swallowing difficulties [9,42]. When performed, laryngoscopy showed vocal cord polyps, nodules, thickening, edema, or paralysis [9,28].

*Ocular.* Eyes and sight are often affected [8,25,28]. Morphological anomalies mostly involve the anterior portion of the eye. Microcornea is identified in half of SMS subjects and iris abnormalities in about 2/3 of them. The iris nodules, once identified as Brushfield-like spots, are more properly definable as Wölfflin-Krückmann spots [13]. The frequency of strabismus was reported to be 55% in a large cohort [25], ranging from 32% [13] to 81% [21] depending on the case series. Refractive defects in SMS include myopia, astigmatism, and hyperopia and should be routinely sought as they may concern up to half of the individuals [21] and be severe [14]. Cataracts and retinal detachments are likely less penetrant [13,25] than previously reported [14]. Nevertheless, the occurrence of retinal detachments may increase with age, being possibly related to severe myopia or self-injurious behaviors [6].

*Musculoskeletal.* As for many other genetic conditions, scoliosis and constipation are common [8,9,21,25,28,43].

*Immunologic.* The production of antibodies is impaired in 60% of individuals, predisposing to recurrent airways infections [44].

*Other* less frequent findings are hypothyroidism, gastroesophageal reflux, hypercholesterolemia, and hypertriglyceridemia [15,25,45].

*Malignancy.* The risk of malignancies is currently considered not to be increased in SMS [6]. However, the typical 17p11.2 deletion comprises the gene *FLCN*, associated with Birt-Hogg-Dubé syndrome (BHDS, OMIM # 135150). This autosomal dominant condition features various cutaneous alterations, spontaneous pneumothorax due to pulmonary cysts, and an increased risk for renal malignancies. A heterozygous pathogenic variant leading to loss-of-function of the *FLCN* gene is required for the diagnosis [46]. Although subjects diagnosed with SMS due to 17p11.2 deletions do not automatically receive an additional diagnosis of BHDS, few anecdotal patients illustrate clinical overlaps between these two conditions [47,48,49,50], raising questions about the opportunity to adopt the oncologic surveillance recommended for BHDS also for SMS.

### 2.4. Neurodevelopmental Features

SMS belongs to genetically determined neurodevelopmental disorders. This condition typically shows the co-occurrence of DD/ID, unique behavioral phenotype, and sleep disturbances.

First concerns about psychomotor development arise by the first year [25]. Hypotonia, lethargy, increased daytime sleepiness and napping, and oromotor dysfunction with reduced vocal production (crying, babbling, vocalizing) are virtually present in all infants. Feeding difficulties are also common and, in the first months, infants often need to be actively awakened during nighttime. This presentation has been summarized in the formula “quiet babies sleeping poorly” whereas the parents might refer to them as “perfect babies” [15,19]. Developmental milestones are delayed. Deambulation is reached on average from 20 to 25 months and first words appear in about two-thirds of individuals by the age of 3 years [25,51]. Speech delay is more severe in the expressive than in the receptive domain, so toddlers may recur to nonverbal communication. Daytime and nighttime toilet training are, respectively, achieved by 78% and 35% of children before thirteen years [25]. The degree of ID ranges from profound to borderline; the majority of subjects score in the moderate ID level [9,25,51,52,53,54,55]. At school, SMS children demonstrate long-term memory, perceptual skills and closure as points of strength while they have relevant weaknesses in short-term memory, sequential processing, and math skills [19,53,55,56,57]. Moreover, scholastic performance is often lowered by maladaptive conduct, hyperactivity, and distractibility [19,58].

The presence of speech delay and stereotypes may recall what is commonly observed in autism spectrum disorder (ASD) [25,51,54]. This overlap is possibly more compelling in toddlerhood, particularly due to poor verbal communication compared to peers [51]. Contrary to what is expected in ASD, in SMS autistic features may be more penetrant in females than in males [59,60]. In a cohort of 20 minors (age range: 4–18) assessed by means of questionnaires addressed to their parents (Social Responsiveness Scale), 90% of subjects scored within ASD, of whom 35% were in the mild/moderate range and 55% were in the severe one [59]. Nevertheless, the subsequent appearance of language, the remarkable communication and socialization skills, and the typical behavioral phenotype may distinguish SMS from typical ASD [54,61]. Another contact point between ASD and SMS is represented by the abnormal processing and modulation of environmental stimuli, including tactile and auditory aversion, hypersensitivity to sounds, vestibular and oral motor dysfunction [15,41,62].

### 2.5. Behavioral Manifestations

Behavioral manifestations are heterogeneous and reflect the integration of self-injurious, stereotypic, and maladaptive components.

Their penetrance and severity are influenced by several determinants, e.g. age, sleep, communicative skills, cognitive level, developmental comorbidities, environmental context, and the underlying genetic defect [8,21,30,51,52,54,58,63,64,65,66,67,68,69]. When the diagnostic process was less straightforward, many subjects were diagnosed with psychiatric disorders, attention deficit hyperactivity disorder (ADHD), or ASD before the diagnosis of SMS was reached [15,59]. Self-injurious behaviors are nearly constant, being observed in more than 95% of individuals [64,69]. The first episodes can be appreciated from the age of 18 months, when repetitive (tilting head, body rocking, rubbing surfaces, excessive mouthing, clapping hand) and self-injurious (self-hitting, biting, or pinching, head banging, hair pulling) behaviors emerge. Onychotillomania (nail yanking) and polyembolokoilamania (insertion of objects into bodily orifices) are specific and strongly evocative of SMS. Onychotillomania and polyembolokoilamania are respectively present in 53.5% and 32.3% of subjects [70] and their occurrence increase with age [63,64]. Polyembolokoilamania can involve ears (31%), nose (17.2%), rectum (3.5%), or vagina (21.1%) [64]. Two additional stereotypies distinctive of SMS are the spasmodic upper-body squeeze (or self-hugging) [71] and the “lick and flip” repetitive page turning [56,63], found in approximately 51% and 46% of patients [63]. Other frequent behavioral problems are teeth grinding, screaming, temper tantrum, hetero-aggressive outbursts, and destruction of property [56,63,70]. On the other hand, adaptative scales show higher scores in communication and socialization skills, both in children and in adults [52,54,66]. This finding reflects the notion of a few individuals with SMS being particularly affectionate, communicative, eager to please but also attention seeking and unceasing talkers [66,67,71]. This attitude coexists with a significant emotional immaturity, which may trigger behavioral responses and make social inclusion difficult. This contrast, termed developmental asynchrony, becomes more appreciable and divergent in those with higher cognitive functioning and in adults: if academic achievements in SMS correspond to the 6- to 8-year-old range, emotional reactions are more in accordance with the 1- to 3-year-old developmental level (the so-called “inner toddler”) [67].

Similarly, daily living skills seem to configure a negative correlation with age, so that in adulthood routine needs appear too demanding in respect of age or, possibly, of ID degree [53,54]. Adults generally rely on their caregivers and only a minority of them are reported to be independent in personal hygiene, cleaning tasks, cooking, or walking short distances in a familiar environment [53].

Eating disorders (foraging and overeating) appear with adolescence [70] and have been found to be directly mediated by *RAI1* haploinsufficiency [27]. These traits are shared with Prader-Willi syndrome, which indeed represents another differential diagnosis due to the presence of infantile hypotonia, short stature, small hands and feet, self-picking, sleep, and behavioral problems [72].

### 2.6. Sleep Disorders

Sleep disorders were mentioned since the first reports of SMS [2,3,33] and their penetrance is nearly complete [8,21,28,73]. The most recurrent features of sleep disturbance are fragmented and shortened sleep cycles, frequent nocturnal and early morning awakenings, and excessive daytime sleepiness [30,73,74,75]. Nighttime sleep may be further deteriorated by the occurrence of enuresis, snoring, and bruxism [73]. Reduced sleep time (on average 1 h less) can be detected by the first year and parents may notice increased somnolence from 12–18 months of age [15]. Interestingly, the circadian rhythm of melatonin secretion is inverted in most SMS patients [30], being active during the daytime rather than nighttime. This reversal, however, is currently not believed to be the only factor responsible for sleep disorders, which are detectable also in individuals with normal melatonin secretion [76,77]. Moreover, the circadian rhythm of body temperature is disrupted, being not inverted but shifted by about three hours in advance [74]. The employment of polysomnography revealed REM sleep to be altered in about half of individuals, resulting mostly reduced but also absent or increased [3,9]. Although sleep disorder persists throughout the entire life, some changes have been identified in the transition toward adulthood. A questionnaire-based study detected an association between older ages and earlier wake-up times, more naps with shorter duration, increased number of nighttime awakenings, and shorter overall sleep time [73]. A subsequent paper employing wrist actigraphy identified an age-related variation in sleep patterns, waking time, progressively getting later, and waking time after sleep onset [74].

### 2.7. Neurological Problems

Seizures are estimated to occur in 11–30% of subjects [3,9]. More recent series substantially confirm this proportion, ranging from 2.1% [25] to 27.5% [8]. An additional 20–25% of individuals develop electroencephalographic abnormalities without a clinical correlation [9,15,78]. No seizure type or electroencephalographic pattern appears to be recurrent, although focal onset impaired awareness seizures (formerly defined complex partial seizures) might occur more frequently [15]; catamenial seizures have been noticed as well [15]. Peculiar neuroradiological findings have been identified in about half of the individuals [9,25], and those more consistent are ventriculomegaly [9,15,25,79,80,81], anomalies of the posterior fossa (enlarged posterior fossa, mega cisterna magna) [9,15,79,80,82], and bilateral decrease in grey matter concentration in lenticulo-insular regions [79]. Several sporadic abnormalities have also been highlighted: unilateral dystrophic calcification of the frontal lobe [9], cortical atrophy [25], hypoplasia of the corpus callosum [25], bilateral periventricular nodular heterotopias [82], hypoplasia of the cerebellar vermis [9,80,81], hypoplasia of the pons [80], enlarged foramen magnum [9], prominent cerebrospinal fluid spaces [9]. Stroke-like episodes have been identified in three subjects, of whom one affected from Moya-Moya disease [29,83,84]; it has thus been recommended to assess cerebrovascular disease in all individuals with SMS before open-heart surgery [84]. The occurrence of peripheral neuropathy, once reported in about 75% of patients [9] and more recently estimated at 38% [25], may produce reduced deep tendon reflexes, a peculiar profile of lower limbs (i.e., the inverted champagne bottle appearance), abnormal gait (flapping feet, toe walking), pes cavus or pes planus [9,15]; moreover, the decreased sensitivity to temperature and pain may worsen the consequences of self-injurious behaviors and polyembolokoilamania [19].

## 3. Genomic and Genetic Cause of SMS

The most common genetic cause of SMS, accounting for about 90% of patients, is an interstitial deletion at 17p11.2, ranging from 1.5 to 9 Mb [5,21]. About 70–80% of the individuals with a 17p11.2 deletion carry a large and recurrent deletion of 3.7 Mb, which results from non-allelic homologous recombination (NAHR) between low-copy-number repeats (LCR) [28,85]. 17p11.2 is a rearrangement-prone genomic region containing seven LCR elements [86]. The genomic instability of this region derives also from the presence of repetitive elements such as Alu elements and AT-rich repeats, that through NAHR and non-homologous end joining (NHEJ) mechanisms are causative of the remaining 20–25% of SMS patients that display atypical deletions of variable size [87].

About 80 known genes map to the 17p11.2 deleted region and for a long time, SMS has been considered a contiguous gene syndrome [3]. Subsequently, the sequence analysis in three patients with SMS phenotype lacking the common deletion disclosed frame-shift variants in *RAI1* (retinoic acid-induced 1), demonstrating its role as the causative gene for SMS [5].

**Figure 2:**
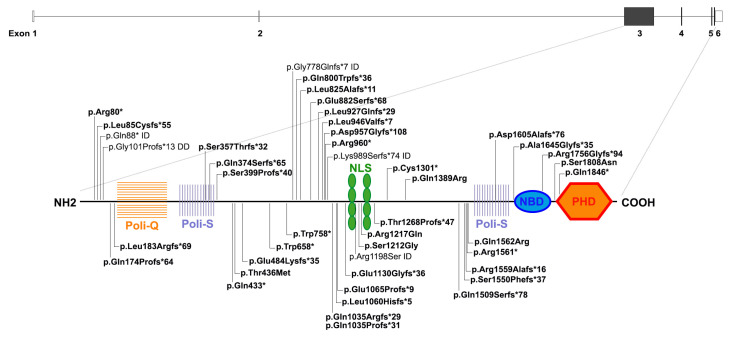
*RAI1* gene and protein structures. At the top, *RAI1* gene structure based on RefSeq NM_030665, including noncoding exons (white) and coding regions (dark grey). At the bottom, *RAI1* protein structure with seven key functional domains, starting with N-terminal a polyglutamine rich tract (Poly-Q, orange), a polyserine rich domain (Poly-S, light purple), a bipartite Nuclear Localization Signal (NLS, green), a second Poly-S tract (light purple), a nucleosome-binding domain (NBD, blue), and a C-terminal “plant homeo-domain” (PHD, red). The pathogenic SMS-associated reported truncating and missense mutations are indicated in bold in the protein structure, the remaining variations reported are not associated with SMS, but with indicated conditions, according to HGMD Professional (version 3 September 2021) and ClinVar database. ID; intellectual disability, DD; developmental delay.

Up to now, about 50 pathogenic variants, mostly non-sense and in-frame located in exon 3 (http://www.hgmd.cf.ac.uk/ac/all.php (accessed on 15 December 2021), HGMD Professional, version 3 September 2021), have been detected in 10% of patients with classical SMS clinical features (Figure 2). All the identified variants result in *RAI1* haploinsufficiency, which is responsible for the SMS phenotype.

A recent study on a large SMS cohort evidenced a higher number of pathogenic variants in *RAI1* than previously reported (23% compared to 10% of all patients with SMS [86]), suggesting that the percentage of *RAI1* variants causing SMS is underestimated [88]. This finding can firstly be explained by the fact that a multisystemic disorder may not be suspected in patients with *RAI1* variants due to the reduced penetrance of congenital anomalies, commonly associated with 17p11.2 deletions. Indeed, notwithstanding the consistent phenotypic overlap among patients harboring either the 17p11.2 classical deletion or a pathogenic variant in *RAI1*, cardiac and renal anomalies, motor delay, short stature, and hearing loss are enriched in carriers of the 17p11.2 deletion [8,21]. Conversely, patients carrying *RAI1* variants show an increased risk of developing overweight/obesity, feeding disorders, polyembolokoilamania, self-hugging, skin picking, muscle cramping, and dry skin [8,11,21]. Secondly, agnostic genetic testing for patients not resembling classical SMS was introduced only recently by means of next-generation sequencing (NGS) technology, which will likely lead to an increased detection of *RAI1* pathogenic variants, with clinical implications for genetic counseling and decision making [89].

SMS is an autosomal dominant condition typically caused by *de novo* deletions or pathogenic variants in *RAI1* at 17p11.2, although familial transmission has been observed [90,91,92]. Zori and collaborators identified a maternal mosaicism for 17p11.2 deletion [90]; other SMS cases of parental mosaicism are known, including one family with three affected sibs due to maternal mosaicism [92]. Even rare complex chromosome rearrangements leading to 17p11.2 deletion have been reported [91,93,94]. In 2017, for the first time, a SMS individual harboring an *RAI1* frameshift variant gave birth to a child with the same genotype [95].

*RAI1* (OMIM *607642, NM_030665) is composed of six exons, four of which are protein coding, and is widely expressed among tissues, including the brain. *RAI1* is highly conserved across different species and is composed of seven different functional domains (Figure 2) [96,97,98,99,100]. Among them there is a C-terminal “plant homeo-domain” (PHD) containing a His-Cys5-His-Cys2-His motif, an extremely conserved motif distinctive of nuclear proteins implicated in chromatin remodeling and in transcriptional regulation [101,102,103]. Indeed, *RAI1* is a transcriptional regulator that enhances the expression of many genes involved in the development and function of the mammalian brain, for instance implicated in the homeostasis maintenance of synaptic plasticity [104] and regulation of circadian rhythm [100,105,106]. 

*RAI1* is classified within epigenetic machinery readers [106] and likely binds to unmodified histone tail H3 through the PHD domain. In addition, proteomic studies suggest that *RAI1* may interact with PHF14, TCF20, HMG20A/iBRAF, which contain several PHD domains as well [107]. Supported by these findings, it was proposed that together they might form a multiprotein complex, the “*RAI1* complex”, functioning as chromatin reader specifically recognizing and binding unmethylated lysine 4 of histone 3 (H3K4) and recruiting MLL1 (KMT2A, OMIM *159555) to tri-methylate H3K4, thereby promoting gene transcription [106]. The biological relevance of these proteins is highlighted by the clinical consequences of their haploinsufficiency, as *KMT2A* and *TCF20* are the causative genes of Wiedemann-Steiner syndrome (WDSTS, OMIM #605130) and of a syndromic neurodevelopmental disorder (OMIM #618430), respectively [108,109].

At the moment, very little is known about the transcriptional regulatory activity and the target genes of RAI1. However, the genome-wide testing approach on SMS-like patients without molecular diagnosis has provided some clues.

## 4. Differential Diagnosis and Related Disorders

**Figure 3:**
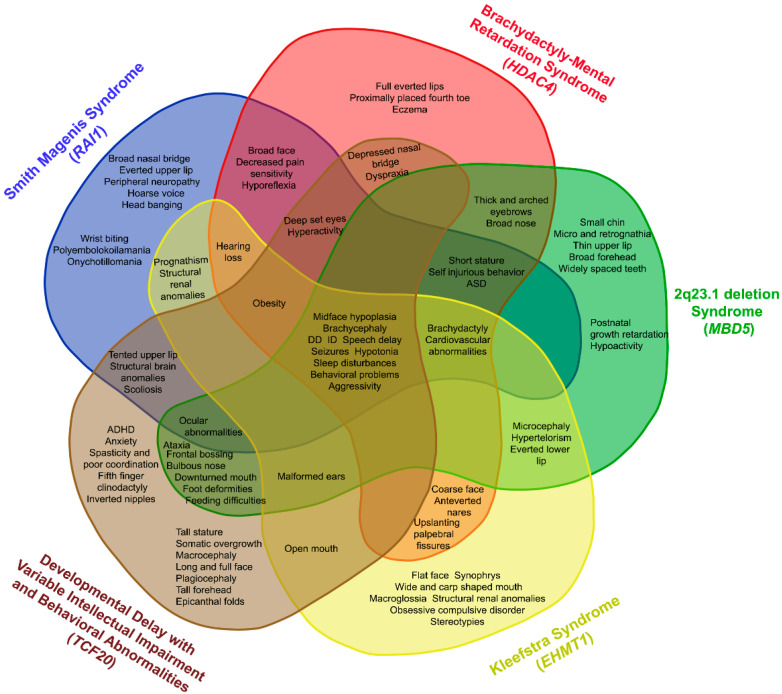
Venn diagram showing common and specific clinical features of SMS-overlapping disorders (2q23.1 deletion, BDMR, KS, and DDVIBA). ID—intellectual disability, DD—developmental delay, ASD—autism spectrum disorder.

Only 50% of individuals with a clinical suspicion of SMS have been confirmed by genetic tests, suggesting that other loci may be directly or indirectly involved in the same pathway of *RAI1*, hence contributing to SMS-like phenotype (Figure 3) [86]. The best approach to discover and understand the molecular basis of complex disorders is by means of genome-wide investigation, such as array-CGH and NGS. Array-CGH screening in SMS-like patients, displaying most of SMS clinical features but lacking either the classical 17p11.2 deletion or *RAI1* mutations, disclosed *HDAC4* and *MBD5* loss-of-function alterations [110,111].

HDAC4 is a class IIa histone deacetylase. Interestingly, quantitative expression analysis on BDMR (BrachyDactyly-Mental Retardation syndrome, OMIM #600430) patients, carrying either a deletion including *HDAC4* or *HDAC4* mutations, resulted in the downregulation of *RAI1* transcripts [110], suggesting that *HDAC4* plays a role as *RAI1* transcriptional regulator. This evidence supports their possible connection and may explain the overlapping phenotypes of SMS and BDMR. The second SMS-related locus is 2q23.1, the region involving the *MBD5* gene [111,112]. Deletions or loss-of-function variants of *MBD5* cause *MBD5*-associated neurodevelopmental disorder (MAND; OMIM #156200). A downregulation of *RAI1* was detected in MAND patients, thus supporting the idea that MBD5 might also exert control on *RAI1* transcription [113]. Moreover, similarly to SMS, MAND patients show sleep disorders and, in patient-derived lymphoblastoid cell lines, *MBD5* haploinsufficiency was demonstrated to lead to the downregulation of clock circadian genes (CCG) (*PER1*, *PER2*, *PER3*, *NR1D2*, *CRY2*) and *RAI1*, thus linking circadian rhythm disruption to *RAI1* expression [114].

In addition, pathogenic *de novo* variants in *MBD5* and in other epigenetic regulators (*SMARCB1*, *NR1I3*, *KMT2C*) were reported in individuals with a clinical diagnosis of Kleefstra syndrome (KS; OMIM #610253) [115,116]. This condition, also known as 9q34.3 deletion syndrome, is due to microdeletions or point variants of the *EHMT1* gene [117]. *EHMT1* encodes a histone methyltransferase, which is considered a histone writer mono- and di-methylating H3K9 (histone 3 lysine 9) and is involved in chromatin remodeling during neurodevelopment and synaptic plasticity [118]. Moreover, the increase of H3K9me2/3 correlates with altered expression of protocadherins, principal regulators of cell-cell adhesion and neuronal connectivity associated with ASD etiology [119]. Although a direct molecular link between *RAI1* and *EHMT1* remains to be proved, the clinical overlap of SMS and KS might be mediated by *MBD5*.

**Figure 4:**
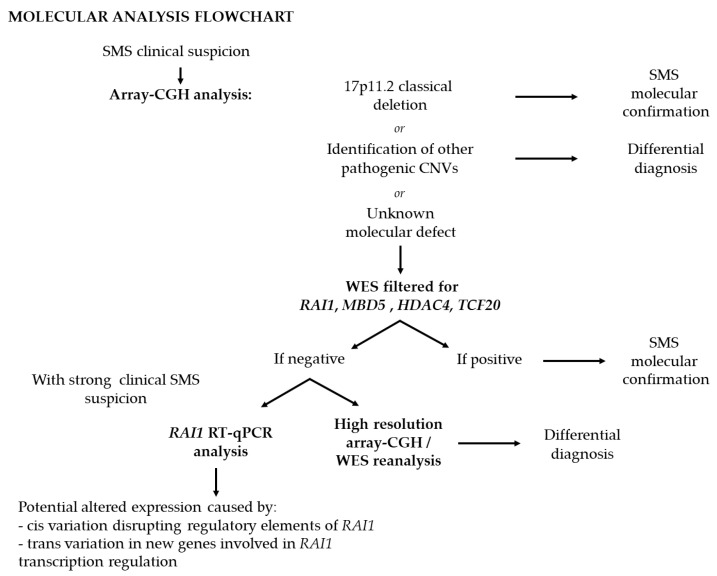
Suggested diagnostic approach for SMS.

Whole-exome sequencing (WES) performed in individuals with a clinical suspicion of SMS but without a molecular diagnosis highlighted potentially deleterious variants in different genes, including *KMTD2*, *MECP2*, *KDM5C, IQSEC2*, and *DEAF1* [120,121]. Notably, a combined approach using WES and array-CGH in 31 unrelated families with an SMS-like phenotype identified pathogenic variants in *TCF20*, defining a new syndrome termed developmental delay with variable intellectual impairment and behavioral abnormalities (DDVIBA, OMIM #618430). These findings support the commonalities in gene structure and function between *TCF20* and *RAI1*, reinforcing the role of the “*RAI1* complex”, of which both are likely part, and likely explaining the shared core clinical features [122]. Based on routine and dedicated genetic analysis, we suggest a diagnostic algorithm for the diagnosis of SMS and SMS-related disorders (Figure 4).

Genome-wide methylation episignatures are significant tools to improve the diagnostic process and provide functional clues. The clinical overlap among SMS and the mentioned conditions does not currently find an epigenetic counterpart, as distinctive episignatures have been detected for *HDAC4*, *MBD5*, *MLL1/KMT2A* but not for *RAI1* [123,124].

Despite these functional and clinical convergences, further studies are needed to explore on different omic levels the functional links among these genes and clarify the biological mechanisms responsible for their phenotypic overlap.

## 5. Treatment Insight

To date, no specific therapy is available for SMS; therefore, its clinical management consists of treating the medical issues presented by each affected individual. Appropriate assessment of related clinical problems, early target intervention, and strict maintenance to therapy can improve overall health, quality of life, and social functioning in SMS patients.

Considering that nearly all SMS individuals have behavioral problems and sleep disturbance, treatments focus on behavioral and educational interventions that target these cardinal traits. Psychotropic medications may be necessary, although evidence regarding pharmacological interventions in SMS is scarce. The array of medications that are prescribed in clinical practice appears to be largely based on clinical experience, case reports, or small series assessing drug efficacy on autism in general and/or ADHD.

According to the retrospective study of Laje et al. [125], stimulants are the preferred medications in the treatment of hyperactivity symptoms. In particular, methylphenidate (MPH) is well-established as first-line treatment with high efficacy and tolerability compared to other psychotropic drugs [126]. Clonidine is sometimes used for the treatment of ADHD in addition or in alternative to stimulants and may have a beneficial effect on sleep in that population [125]. Among the antipsychotics, risperidone shows a marked improvement of symptoms of hyperactivity and/or maladaptive behaviors (including irritability, aggression, outbursts, and self-injury) [127,128]. Adverse effects of risperidone include weight gain, increased appetite, hyperlipidemia, insulin resistance, fatigue.

Furthermore, the management of dysfunctional sleep patterns should also be considered as a key clinical goal in SMS subjects. Good sleep hygiene should include maintaining regular day and night routines, developing rituals that help with relaxation, keeping the sleep environment dark during the night, pleasant and relaxing, and avoiding caffeine and electronic devices. However, pharmacotherapy is often required to optimize the routine management of sleep disorders. Melatonin is a common nonprescription pharmacologic treatment particularly appropriate for populations with sleep disturbance due to circadian phase delay, as for SMS. Evidence to support the use of short-acting melatonin supplementation consists of small case series and anecdotal reports by SMS parents. Overall, short melatonin treatment trials reported a significant decrease in sleep latency with little impact on the total duration of sleep or behavioral manifestations. Optimal melatonin dosing ranged from 0.75 to 10 mg/day taken approximately 90 min before bedtime [129,130]. The combined regimen of nocturnal melatonin and daytime administration of acebutolol, an adrenergic antagonist that blocks melatonin secretion, proved to be beneficial. This strategy may increase nocturnal melatonin concentrations, improve nocturnal sleep and behavior, and aid in correcting sleep patterns [131].

Tasimelteon is an oral melatonin receptor agonist that was developed for the treatment of circadian sleep–wake rhythm disorders. In 2020, this drug was approved in the US for the treatment of nighttime sleep disturbances in SMS [132]. Ramelteon, another melatonin agonist approved for the treatment of insomnia, has not been studied specifically in patients with SMS but has been shown to shift the circadian phase in individuals with jet lag [133] and might be effective also in SMS.

Other drugs that could be useful in the management of sleep problems in children with SMS include antihistamines (i.e., diphenhydramine), antidepressants (i.e., trazodone), atypical antipsychotics (i.e., quetiapine), gabapentin, or other anticonvulsant drugs (appropriate for children with concomitant seizure disorders) [129].

## Data Availability

Data sharing not applicable.
